# Mapping Regulatory Determinants in Plants

**DOI:** 10.3389/fgene.2020.591194

**Published:** 2020-10-28

**Authors:** Mary Galli, Fan Feng, Andrea Gallavotti

**Affiliations:** ^1^Waksman Institute of Microbiology, Rutgers University, Piscataway, NJ, United States; ^2^Department of Plant Biology, Rutgers University, New Brunswick, NJ, United States

**Keywords:** plant genomics, transcriptional regulation, chromatin, transcription factor binding, cis-regulatory regions

## Abstract

The domestication and improvement of many plant species have frequently involved modulation of transcriptional outputs and continue to offer much promise for targeted trait engineering. The cis-regulatory elements (CREs) controlling these trait-associated transcriptional variants however reside within non-coding regions that are currently poorly annotated in most plant species. This is particularly true in large crop genomes where regulatory regions constitute only a small fraction of the total genomic space. Furthermore, relatively little is known about how CREs function to modulate transcription in plants. Therefore understanding where regulatory regions are located within a genome, what genes they control, and how they are structured are important factors that could be used to guide both traditional and synthetic plant breeding efforts. Here, we describe classic examples of regulatory instances as well as recent advances in plant regulatory genomics. We highlight valuable molecular tools that are enabling large-scale identification of CREs and offering unprecedented insight into how genes are regulated in diverse plant species. We focus on chromatin environment, transcription factor (TF) binding, the role of transposable elements, and the association between regulatory regions and target genes.

## Regulatory Regions and Mechanisms Revealed by Classic Studies

**Figure 1:**
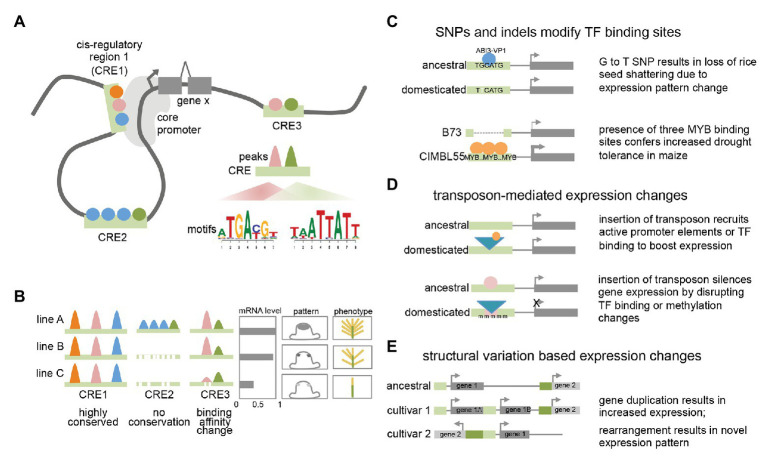
Plant transcriptional regulation **(A)** model of plant transcriptional regulation at gene X. Colored circles represent different TFs binding to three distinct cis-regulatory regions (CREs; light green bars) that can contact the core promoter *via* DNA looping. Motifs enriched within binding peaks for two TFs are shown for CRE3. **(B)** Conservation and variation of TF binding events among different lines or accessions. Colored peaks represent different TF binding events within CREs. mRNA expression levels, cell-type specific expression pattern, and resulting phenotype are shown. **(C)** Examples showing how single nucleotide polymorphisms (SNPs) and indels can result in expression and phenotypic changes. **(D)** Examples showing how transposon insertions can result in expression and phenotypic changes. **(E)** Examples showing how structural variants can result in expression changes.

Mining trait-associated genetic factors has traditionally been performed using classical genetics, GWAS, and QTL analysis. Examples from these studies serve as excellent guides for understanding the molecular basis of phenotypic diversity (Deplancke et al., 2016). In particular, the regions corresponding to several beneficial traits associated with the domestication and diversification of many plant species from their wild relatives have been mapped by these approaches and frequently shown to be located in the intergenic space, sometimes residing up to 100 kb from the closest protein coding genes (Figure 1A; Olsen and Wendel, 2013; Rodgers-Melnick et al., 2016; Swinnen et al., 2016; Lu et al., 2019). Correspondingly, these traits involve variations in gene expression, with variants affecting either the level of expression or the spatial and/or temporal pattern of expression of certain genes (Figure 1B; Meyer and Purugganan, 2013; Springer et al., 2019). Unlike changes to protein-coding genes which often result in easily interpretable loss-of-function alleles, the exact causative features underlying functional cis-regulatory regions (CREs) are currently difficult to identify given the variable nature of regulatory elements, their frequent gene-distal location, and the lack of an obvious rigid code that determines their functionality. Understanding the molecular nature of these changes however lies at the heart of our ability to accelerate crop improvement using CRISPR-based targeted engineering of useful traits and traditional breeding (Rodríguez-Leal et al., 2017; Chen et al., 2019; Eshed and Lippman, 2019; Springer et al., 2019).

In several cases, the molecular nature of the phenotypic variation has been determined and found to be associated with a range of different causes. These include single nucleotide polymorphisms (SNPs) that affect transcription factor (TF) binding, either by disrupting or recruiting additional TF binding sites. For example, a G to T nucleotide change located 12 kb upstream of the *qSH1* gene in rice, a BEL-type homeobox TF, is believed to disrupt an ABI3-VP1 TF binding site (Konishi et al., 2006). This results in a loss of *qSH1* expression in the pedicel abscission zone and a subsequent non-shattering phenotype that facilitated higher harvesting yields. Alternatively, changes in TF binding can also involve advantageous gain of function elements. A GWAS screen for drought tolerance in maize identified a 366 bp region located in the proximal upstream region of *ZmVPP1*, a vacuolar-type H^+^-pyrophosphatase, that conferred increased drought tolerance in several varieties (Wang et al., 2016). This fragment contains three putative MYB binding sites, which were shown to increase expression of *ZmVPP1* relative to the drought-sensitive maize line B73, which lacks the MYB binding sites.

In other cases, functional traits associated with cis-regulatory elements (CREs) may not involve nucleotide variations that directly correspond to known TF binding sites but are instead located nearby. This is the case for the rice *GW7* gene, which affects grain width and grain quality (Wang et al., 2015b). Certain rice varieties were found to contain two short indels directly adjacent an SBP16/GW8 TF binding motif in the proximal upstream region of *GW7*. These indels do not directly disrupt the TF binding motif but do appear to lower expression of *GW7* relative to varieties in which the indels are absent. Given that regulatory regions typically contain multiple different TF binding sites (Hardison and Taylor, 2012; Ricci et al., 2019), such examples could indicate that these divergent regions simply correspond to unknown TF binding sites and reflect the incompleteness of TF binding motif characterization in plants. Alternatively, they could alter local DNA shape (i.e., the sequence-dependent DNA structure surrounding the motif) or spacing between adjacent motifs, among other factors that contribute to the complexity of TF binding specificity (Slattery et al., 2014). Such examples highlight the need for comprehensive annotation of TFs and other regulatory regions. Similar examples have been noted in non-plant studies, where there is accumulating evidence that causative SNPs frequently do not directly affect TF binding motifs, but may impact cooperative or collaborative binding of TF complexes (Deplancke et al., 2016).

Transposon insertions in regulatory regions can also influence gene expression of adjacent genes, resulting in either elevated or suppressed gene expression levels, and likely act through a variety of mechanisms (Hirsch and Springer, 2017; Zhao et al., 2018). A classic example of the former in plants is the presence of a *Hopscotch* element located ~60 kb upstream of the *TEOSINTE BRANCHED1 (TB1)* gene, a TCP-family TF that determines the apical dominance of domesticated maize relative to its highly branched wild ancestor teosinte (Studer et al., 2011). The *Hopscotch* element enhances the expression of *TB1* through an unknown mechanism. Interestingly, a nearby *Tourist* transposon within the same enhancer appears to repress expression of *TB1*, highlighting the dynamic nature of transcriptional changes conferred by transposable elements. Another illustrative example includes the insertion of a *Copia* retroelement in the proximal upstream region of the *RUBY* gene in blood oranges. *RUBY* encodes a MYB TF involved in anthocyanin production and its expression level is increased by cold-induced expression conferred by sequences within the long terminal repeat (LTR) that are hypothesized to harbor either promoter-like features with a TATA box and TSS, or other upstream activating sequences (Butelli et al., 2012). These examples suggest that like other cases from animals, transposons may act as novel promoters by recruiting the basal transcriptional machinery or introducing tissue-specific TF binding sites (or disrupting repressive TF binding sites; Butelli et al., 2012; Sundaram et al., 2014).

Transposon insertions within regulatory regions are also able to negatively impact gene expression. They can do this by disrupting existing TF binding sites or other regulatory features, or *via* epigenetic changes typically involving repressive DNA methylation (Huang and Ecker, 2018). For example, one of the major factors determining fruit color in grape species, is caused by a *Gypsy-like* retrotransposon insertion, *Gret1*, in the upstream region of *MYBA1*, involved in berry anthocyanin production. As opposed to the *RUBY* blood orange case described earlier, the presence of *Gret1* results in loss of gene expression and the white-colored berries typical of chardonnay (Kobayashi et al., 2004). Similar cases of transposon mediated gene repression are also seen in maize at the *ZmCCT10* and *ZmCCT9* loci, two genes involved in flowering-time regulation whose causative transposon insertions reside 2.5 and 57 kb upstream, respectively (Yang et al., 2013; Huang et al., 2017). In general, the mechanisms of how such transposon associated CREs influence expression are not fully understood although these examples and others suggest they can affect both distal enhancers and proximal regulatory regions. In other cases involving transposon insertions in regulatory regions, changes in DNA methylation have been documented as the underlying cause of stable gene downregulation (Hirsch and Springer, 2017). Examples of such epialleles include a methylated *hAT* element inserted in the proximal regulatory region of the melon *CmWIP* gene, which controls sex determination (Martin et al., 2009) and a SINE retrotransposon inserted upstream of the tomato *VTE3* gene, involved in vitamin E biosynthesis (Rossi et al., 2014). Possible mechanisms that explain stable transposon-triggered repression include spreading of methylation marks from the TE into the adjacent regulatory region, thus altering chromatin accessibility or blocking TF motif binding (many TFs preferentially bind unmethylated sites; Eichten et al., 2012; O’Malley et al., 2016; Huang et al., 2018). Overall, these examples as well as studies analyzing global transposon location (i.e., 86% of maize genes contain a TE within 1 kb of the gene; Hirsch and Springer, 2017) and association with eQTL, suggest that TE-driven transcriptional influence is frequent and in certain genomes may be major drivers of regulatory variation (Zhao et al., 2018; Noshay et al., 2020).

Although far less frequent than regulatory changes associated with TE insertions, there are several reports of regulatory epialleles that appear to have formed spontaneously. These include the *Colorless non-ripening (Cnr)* mutant allele of tomato, which encodes an SBP TF that affects color ripening (Manning et al., 2006). In the *Cnr* mutant, the upstream regulatory region of the *Cnr* gene is stably hypermethylated throughout development, leading to reduced expression of the gene (Zhong et al., 2013). Interestingly, the methylated sites are adjacent to two MADS-box TF binding sites bound by RIPENING INHIBITOR1 (RIN1; a MADS-box TF) in ChIP-seq (Zhong et al., 2013) suggesting that methylation changes in the *Cnr* epimutant could impact TF binding.

Finally, structural variants have also been shown to affect regulatory outputs by altering gene copy number and/or the arrangement or composition of CREs (Alonge et al., 2020), highlighting the modular architecture of regulatory elements. In the case of inversions, a certain gene may become located adjacent to an otherwise distally located gene or regulatory region and assume novel expression patterns. This appears to be the case for the classic *Tunicate* allele of maize, which shows unusually long glumes in both inflorescences as a result of ectopic expression from the 3' region of a gene normally located 1.8 Mb away (Han et al., 2012). Other structural variants include segmental duplications that increase gene copy number. While these do not directly involve changes in CREs, they do appear to be a subtle but possibly frequent mechanism of trait-associated transcriptional modulation in certain species (Alonge et al., 2020). Other situations in which putative regulatory regions are rearranged or duplicated are less clear. A good example of this is the ~4 kb DICE distal enhancer element in maize which confers increased expression of the *BX1* gene and consequently increased herbivore resistance (Betsiashvili et al., 2015; Zheng et al., 2015). The DICE element appears to be a divergent duplication of nearby sequences, and the increased expression may result from increased recruitment of specific TFs (Galli et al., 2018). Additional examples from maize include the classic cases of the *b1* and *Vgt1* loci, both of which are associated with structural variation in distal non-coding regions that results in epigenetic changes (Stam et al., 2002; Castelletti et al., 2014).

Detailed genetic and molecular characterization of QTL and classic cases have established a solid groundwork for understanding how regulatory changes influence many phenotypic traits in plants. However, they likely represent only a small fraction of the genetic variation and molecular mechanisms that govern transcriptional response for quantitative traits. Recently-developed genomics based techniques are paving the way for large-scale mining of putative CREs and begin to outline certain molecular signatures that correlate with gene expression and are conserved across species and accessions (Maher et al., 2018; Lu et al., 2019; Alonge et al., 2020). Ultimately, combining both genetic and genome-wide studies will prove a powerful technique to better understand beneficial traits.

## Genome-Wide Identification of cis-Regulatory Regions

Regulatory DNA in eukaryotes is generally characterized by chromatin accessibility, low DNA methylation, and is often associated with distinct histone modifications (Marand et al., 2017; Oka et al., 2017; Klemm et al., 2019; Lu et al., 2019). In plants, several recent studies have taken advantage of these properties to mine candidate regulatory elements at the genomic level (Sullivan et al., 2014; Rodgers-Melnick et al., 2016; Oka et al., 2017; Lü et al., 2018; Maher et al., 2018; Lu et al., 2019; Ricci et al., 2019; Parvathaneni et al., 2020). Such approaches are critical because while previous promoter and QTL studies suggest that most regulatory elements appear to lie within 1–2 kb upstream of the gene body in smaller genomes such as *Arabidopsis*, in larger genomes, regulatory regions reside within a much broader upstream area, with distal elements occasionally located hundreds of kb from the genes they regulate, making their identification by traditional means arduous. Therefore, the identification of accessible chromatin regions (ACRs) using techniques such as ATAC-seq (Assay for Transposase-Accessible Chromatin using sequencing), MNaseHS (micrococcal nuclease hypersensitivity), and DNaseHS (DNAse hypersensitivity) has been highly informative for mapping regulatory regions in plants, revealing their frequency, size, and location, as well as many other important aspects. These studies demonstrate that ACRs are most often found near transcription start and end sites, but can also frequently be found over 2–200 kb from any gene depending on the species (Sullivan et al., 2014; Rodgers-Melnick et al., 2016; Oka et al., 2017; Maher et al., 2018; Lu et al., 2019; Ricci et al., 2019). They also show that ACRs can be condition and tissue-specific, highlighting the dynamic nature of chromatin (Sullivan et al., 2014; Rodgers-Melnick et al., 2016; Oka et al., 2017; Maher et al., 2018; Ricci et al., 2019; Parvathaneni et al., 2020). In support of their functionality, most identified ACRs are enriched for TF binding events and motifs and show transcriptional enhancer activity (see below for more detail; Sullivan et al., 2014; Ricci et al., 2019). Importantly, it was shown that SNPs in ACRs explain up to 40% of the variability in quantitative traits in maize and in particular overlap with several classically defined distal QTL discussed previously, substantiating their functionality and highlighting the role of regulatory regions in modulating phenotypes (Rodgers-Melnick et al., 2016; Ricci et al., 2019).

A landmark, cross-species comparative study of 13 angiosperm species with genome sizes ranging from ~100 to 5,000 Mb demonstrated that ACRs account for 0.2–6.5% of the total genome of a species and that their location varies according to genome size (Lu et al., 2019). For example, while the total sequence length of ACRs was fairly consistent across species regardless of genome size, large genomes showed a greater percentage of distally located ACRs (i.e., small genomes such as *Arabidopsis* showed that only ~6% of all ACRs were distal compared to ~46% in barley). Transposon insertions were found to be one of the main factors contributing to this occurrence, presumably pushing ACRs away from genes (Lu et al., 2019). Transposons themselves also appeared to be responsible for creating certain species-specific distal ACRs, as noted previously from classical studies (see above i.e., maize *TB1*). The controlled parallel nature of the Lu et al. (2019) study also allowed several important cross-species observations such as the finding that the number of ACRs correlated with the number of genes within a species and that many distal ACRs were conserved between sister species. Overall, an important finding from this study is that large and small plant genomes appear to be structured differently, despite harboring many of the same genetic pathways and gene regulatory networks (Lu et al., 2019). This underscores the importance of empirically mining sufficient amounts of regulatory information both for direct application in a species of interest such that ultimately such information will enable accurate machine learning predictions in other crop species.

A major factor in the characterization of putative regulatory regions is determining their functionality. In animals, regulatory regions are generally categorized into classes such enhancers, insulators, or promoters depending on their role in gene expression (Andersson and Sandelin, 2020). These terms however remain somewhat ambiguous despite an enormous effort toward their classification, perhaps because the elements themselves are heterogeneous (Andersson and Sandelin, 2020; Gasperini et al., 2020). In plants, these operational definitions are even more vague; however, studies have begun to tease out some common trends. Plant adapted versions of massively parallel promoter and enhancer reporter assays such as self-transcribing active regulatory region sequencing (STARR-seq; Ricci et al., 2019; Jores et al., 2020), show that many ACRs are capable of enhancing gene expression (Ricci et al., 2019). Traditional STARR-seq works by inserting fragments either from randomly sheared genomic sequence, BAC libraries, or small fragments such as those from ATAC-seq and placing them downstream of a cassette containing a minimal promoter fused to GFP (Arnold et al., 2013). Because enhancers are assumed to be capable of controlling gene expression regardless of distance or orientation (according to the classical definition), STARR-seq allows for self-driven transcription of the element and quantitative readout. In maize, both proximal and distal ACRs were found to show a general enhancement of activity, relative to randomly selected regions with similar features (Ricci et al., 2019). On the other hand, a modified version of STARR-seq using transient transfection in tobacco leaves found that four known plant enhancers gave the strongest transcriptional output when placed immediately upstream of a minimal promoter and were not active when placed in the 3'UTR of the reporter gene (Jores et al., 2020). Further studies are needed to tease out the functional determinants and optimal architecture of the various classes of regulatory elements. Given their utility to generate synthetic transcriptional units for agricultural improvement (Liu and Stewart, 2016), findings from such assays, and approaches could be directly applicable in plants, unlike in animals.

Genomes also typically harbor specific chromatin features that serve as another potential source of regulatory information (Marand et al., 2017). In animals, ACRs are often associated with distinct histone modifications that correlate with gene expression outputs (Hardison and Taylor, 2012; Gasperini et al., 2020). There has been much focus placed on using unique signatures of these various chromatin marks to identify particular classes of regulatory elements (e.g., enhancers) to aid genome annotation efforts and understand how chromatin environment impacts gene expression. However, it is widely accepted that operational definitions based on these biochemical marks serve as a guide rather than a fixed rule (Gasperini et al., 2020). Several large-scale studies have profiled histone modifications in various plant species (Oka et al., 2017; Lü et al., 2018; Lu et al., 2019; Peng et al., 2019; Ricci et al., 2019), and detailed analysis suggests that as in animals, certain chromatin signatures correlate with gene expression levels: expressed genes are enriched for H3K4me3, H3K56ac, and H2A.Z at the transcription start site, whereas repressed genes are enriched for H3K27me3 and H2A.Z (Lu et al., 2019). Furthermore, in maize, it appears that H3K27me3 marks often correspond to tissue-specific genes while H3K4me1 and H3K4me3 tend to mark broadly expressed genes (Lu et al., 2019; Peng et al., 2019; Ricci et al., 2019). Combining histone modification data with ACRs found that H3K9/K27/K56ac marks were generally associated with high expression levels of nearby genes and may represent enhancers. Distal ACRs instead marked by H3K27me3 tended to be located near genes with lower levels of expression and may represent repressor elements. Interestingly, it appears that some plant histone modification trends differ from those found in animals (Lu et al., 2019). For example, while H3K4me1 marks are typically found at distal CREs, in plants, this modification was not frequently associated with distal CREs (Lu et al., 2019).

Finally, DNA methylation maps are also highly valuable for mining regulatory information (Crisp et al., 2019). Prior studies have noted that most ACRs are hypomethylated, and in large genomes that are typically heavily methylated, unmethylated regions (UMRs) serve as an excellent tool to mine functional CREs (Crisp et al., 2020). Importantly, UMRs tend be static across most tissues and conditions in plants, whereas ACRs and histone modifications are often dynamic. Therefore, UMRs from a single tissue can be used to locate CREs, and when paired with chromatin accessibility data from a dissimilar tissue, can reveal CREs potentially set to become accessible or expressed in another tissue (Crisp et al., 2020).

**Figure 2:**
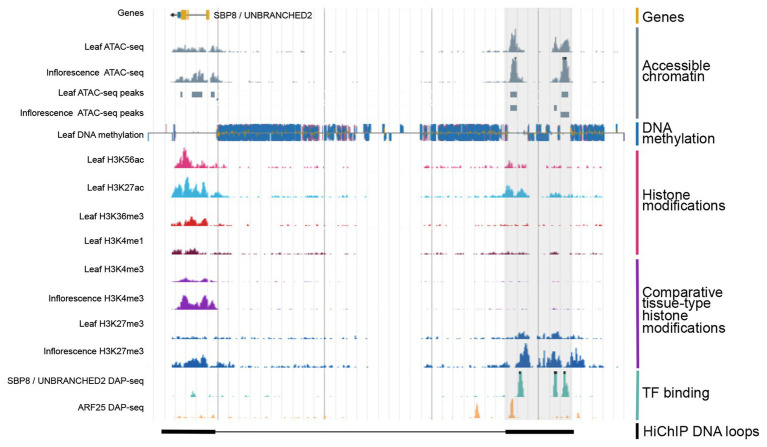
Integration of various types of genomic regulatory data allows for the identification of CREs. Shown is a genome browser view of putative distal CRE (gray shaded region) located 40 kb upstream of the SBP8/UNBRANCHED2 gene in maize. Data obtained from Ricci et al., 2019.

Overall, these various genome-wide approaches for mining regulatory elements are generating highly informative maps that are crucial for understanding regulatory dynamics (Figure 2). Such data are critical for locating regulatory regions for use in transgenic studies or harnessing tissue-specific promoters for genetic engineering purposes.

## Transcription Factors: Drivers of Gene Expression

At the heart of transcriptional regulation is DNA-binding TFs and TF complexes bound to CREs. Transcription factors recognize short DNA sequence motifs in regulatory regions of their target genes and control the gene expression changes responsible for plant developmental programs and environmental responses. TFs bind to family-specific DNA motifs that contain four to six nucleotides, although many instances of longer and more complex architecture are known (Jolma et al., 2013; Weirauch et al., 2014; O’Malley et al., 2016). Particularly, in the case of short motifs, it is clear that TFs do not bind to all instances of these motifs within a given genome, suggesting that other factors also influence binding specificity (Hardison and Taylor, 2012; Todeschini et al., 2014). These have been shown to include DNA shape, i.e., the DNA sequence surrounding the motif, which is not directly bound by the TF (Slattery et al., 2014), as well as other factors such as the presence of proximally located motifs that can be bound by cooperating TFs (Deplancke et al., 2016). However, while these features play a role, the precise determinants of TF binding specificity remain unclear. One of the many additional interesting features of TF binding is the tendency for diverse TFs to bind in clusters, often lying within a region of open chromatin (Figure 1; Gasperini et al., 2020). This has been observed in many animal systems where a large number of genome-wide TF binding maps are available, and appears to occur in plants as well (see below for more detail). It remains unclear how these clusters of TFs are involved in gene regulation; however, the modular/combinatorial binding nature of these regulatory regions (i.e., multiple TFs binding) appears to allow genes to be controlled in tissue-specific or temporal manner (Spitz and Furlong, 2012). In plants, this is particularly intriguing from an agronomic engineering perspective because it suggests that phenotypes associated with distinct organs (i.e., ear traits but not tassel traits in maize) could be separated, allowing specific alterations to one organ or conditional response without altering another with a less desirable phenotype (Dong et al., 2019).

There are several methods by which to identify TF binding. ChIP-seq is the current gold-standard method for determining *in vivo* binding sites of TFs in live cells (Johnson et al., 2007). This method enables the identification of genomic binding sites in a tissue-specific chromatin context with high resolution (Park, 2009; Kaufmann et al., 2010). DNA-protein complexes are immunoprecipitated using an antibody specific to the protein of interest or a tag that is fused to the protein, and DNA is purified from the immunoprecipitated complex and subjected to next-generation sequencing. Several key factors that contribute to high-quality data in ChIP-seq, include antibody selection, negative controls, and biological replicates (Park, 2009; Kidder et al., 2011; Landt et al., 2012). Because of its *in vivo* context, ChIP-seq captures DNA bound both directly and indirectly by the TF of interest. This can include sites bound by hetero- or multimeric complexes. Many small and medium scale ChIP-seq studies have been carried out in *Arabidopsis* in contrast to the handful that have been performed in larger genomes such maize and soybean (Bolduc et al., 2012; Huang et al., 2012; Gregis et al., 2013; Lau et al., 2014; Tsuda et al., 2014; Li et al., 2015; Pautler et al., 2015; Jung et al., 2016; Song et al., 2016; Feng et al., 2018; Jo et al., 2020). A major limitation to ChIP-seq in plants is the time and effort required to either create transgenic lines or generate antibodies.

Performing ChIP-seq using protoplasts that transiently express epitope-tagged transcription factors is an alternative approach (Kong et al., 2012; Lee et al., 2017; Tu et al., 2020), as in some cases, specific antibodies against an endogenous protein of interest or transgenic lines expressing the protein of interest fused with a tag in a mutant background are unavailable. Protoplasts can be obtained either from mesophyll or other tissues such as root or stem and are transformed with a plasmid that expresses the protein of interest fused with an epitope-tag driven by a ubiquitously expression promoter such as 35S (Hernandez et al., 2007; Yoo et al., 2007; Kong et al., 2012; Para et al., 2014). ChIP-seq using protoplasts has obvious advantages as it bypasses the requirements for antibody or transgenic plants; however, overexpression of proteins in protoplasts might lead to altered genomic binding profiles due to excess protein in the cell (Kidder et al., 2011). A recent large-scale study using this approach in maize to map the binding sites of 104 TFs in leaves observed several key findings. As seen in animals, plant TF binding sites clustered together, covering ~2% of the maize genome and reinforcing the emerging paradigm that multiple TFs are needed for regulation of a single locus (Tu et al., 2020). These results also suggest co-binding appears to be important for TF specificity in maize (Tu et al., 2020).

Another modified version of ChIP-seq is cleavage under targets and release using nuclease (CUT&RUN), a chromatin profiling strategy in which antibody-targeted controlled cleavage by micrococcal nuclease releases specific protein-DNA complexes into the supernatant for paired-end DNA sequencing (Skene and Henikoff, 2017; Skene et al., 2018). Compared to ChIP-seq, CUT&RUN has several key advantages such as no crosslinking, which avoids false positive signals; *in situ* targeted digestion, which greatly reduces background; efficiency, as it can be finished in a day; and high signal-to-noise ratio, requiring only one tenth of the sequencing depth as ChIP-seq.

DAP-seq is an *in vitro* alternative to ChIP-seq (O’Malley et al., 2016). DAP-seq works by combining a standard Illumina-based genomic DNA sequencing library together with an *in vitro* expressed affinity-tagged TF coupled to magnetic beads. After a series of washes, TF-bound DNA is eluted, enriched, and barcoded for multiplexing, followed by next-gen sequencing (Bartlett et al., 2017). Resulting reads produce genome-wide peak maps similar to ChIP-seq, but often with higher resolution. A main advantage of DAP-seq is that it combines the low cost and high throughput of an *in vitro* assay with DNA in its native sequence context thereby preserving DNA structure and DNA methylation marks that are known to impact TF binding (O’Malley et al., 2016). Bound fragments are directly mapped to a genome unlike other *in vitro* assays such as HT-SELEX and protein binding microarrays, which report only motifs (Jolma et al., 2013; Weirauch et al., 2014). DAP-seq has been used to generate high quality peak maps for 529 *Arabidopsis* TFs and several maize TFs (O’Malley et al., 2016; Galli et al., 2018; Ricci et al., 2019). This data revealed many informative properties of plant TFs such as high frequency at which TFs from the same family- or subfamily-type bind similar sites, that TFs bind a very small fraction of all motif instances, and again that TFs cluster together in proximal promoters (and distal enhancers which are often located over 20–100 kb from their putative target gene in maize). Comparative studies of DAP-seq showed significant overlap with ChIP-seq data; however, DAP-seq generally produces more peaks than ChIP-seq suggesting that DAP-seq captures binding events that take place independent of tissue- or condition-specific chromatin information (O’Malley et al., 2016).

Genome wide TF binding maps generated by these various techniques will be essential for understanding factors influencing both TF binding and TF activity. Yet while TFs are the major modulators of transcriptional activity, and their individual importance is often evident from mutations with severe developmental consequences, how TFs actually modulate gene expression remains largely unclear (de Boer et al., 2020). As in animal systems, it is also clear that not all TF binding is functional (Spitz and Furlong, 2012; Para et al., 2014; Brooks et al., 2019; Gasperini et al., 2020). Therefore, another challenge will be establishing determinants of TF activity and how these are influenced by factors such as position of binding sites, binding site strand, helical position, and protein interactions (de Boer et al., 2020). As mentioned previously, TF binding sites often cluster together and form cis-regulatory modules (CRMs; Hardison and Taylor, 2012) which themselves could impact TF activity. These CRMs and the individual TF binding sites within are often conserved within and across species indicating that together they may be important for TF activity and gene expression. Deciphering the degree to which plant TFs may work cooperatively will require dissection of CRMs using both natural variation and targeted genomic editing to better understand these regulatory regions.

## Interactions Between Regulatory Regions and Genes: Target Gene Identification and Functional Consequences of 3D Conformation

An essential aspect of mining regulatory elements in any genome is being able to associate a putative regulatory region with a target gene or genes, and its expression dynamics. This remains a particularly challenging task in large genomes where regulatory regions may be located hundreds of kb away (Pliner et al., 2018). The current model of regulatory region-gene interactions involves looping of DNA in 3D space to allow physically distant regions to contact core promoters (Figure 1A; Shlyueva et al., 2014), and until recently this general eukaryotic model was derived largely from data in animals. Several plant studies using chromosome conformation capture (3C)-based techniques such as Hi-C and other variants, which capture global chromatin interactions (van Steensel and Dekker, 2010), have now shown that plant 3D chromatin organization generally resembles that reported in animals (Wang et al., 2015a, 2017; Dong et al., 2017; Liu et al., 2017; Mascher et al., 2017; Li et al., 2019; Peng et al., 2019; Ricci et al., 2019; Sun et al., 2020), despite the absence of certain proteins such as CTCF that are associated with this phenomenon in animals (Liu et al., 2017; Rowley et al., 2017). In these assays, chromatin contacts within a particular tissue are first cross-linked with formaldehyde, sheared to linearize the DNA, and then DNA ends are ligated together. The resulting ligated DNA is sequenced and consists of fragments that may not reside close in linear genomic space but are contacted in 3D space, often reflecting long-range spatial associations. Importantly, comparison among various plant genomes suggests that the 3D architecture of small, compact plant genomes such as *Arabidopsis* which tend to have CREs located within or near genes, differs from that of larger plant genomes which often form extensive long-range chromatin loops (Wang et al., 2015a, 2017; Dong et al., 2017; Liu et al., 2017; Ricci et al., 2019).

Bulk chromatin capture techniques such as Hi-C are often limited in their resolution, preventing the detailed empirical mapping of linkages between regulatory regions and target genes, and thus limiting the functional mapping of regulatory elements. More focused techniques such as Hi-ChIP and ChIA-PET use antibodies to enrich for a specific subset of chromatin interactions that are associated with RNA polymerase II, a particular histone modification, or transcription factor, offering greater resolution at a lower sequencing depth (Fullwood et al., 2009; Mumbach et al., 2016). A series of reports that mapped 3D chromatin interactions using several different higher-resolution assays in maize, a model species that is likely representative of many large crop genomes, revealed the importance of chromatin loops for influencing gene expression and phenotype (Li et al., 2019; Peng et al., 2019; Ricci et al., 2019; Sun et al., 2020). Collectively, these studies indicated that: (i) interactions between genes and proximal (<2 kb) and distal (>20 kb) ACRs (i.e., putative CREs) were common, and confirmed many genetically identified long-distance regulatory regions; (ii) genes with chromatin interactions associated with active promoters and enhancers tended to have higher expression levels than those without; (iii) functional CRE-gene interactions showed a strong loop signal intensity and tended to lie directly upstream of the gene (i.e., gene skipping was less common than direct contact; Ricci et al., 2019); (iv) gene pairs connected by loops within their proximal promoters were often transcriptionally coordinated; (v) tissue-specific (i.e., ear vs. shoot) proximal-distal interactions correlated with tissue-specific gene expression; and (vi) genes and CREs were often connected by multiple loops suggesting a complex pattern of regulation. Many of these features are likely to be conserved in other plant genomes and serve as a foundation for predicting functional regulatory elements in other species. However, given the vast diversity and size differences among plant genomes, and the prevalence of polyploidy among domesticated crop species, it is possible that many species exhibit unique chromatin conformation features that influence gene expression and certain species-specific traits (Wang et al., 2017; Concia et al., 2020).

Overall, these studies in plants confirm that long-range contacts do frequently occur in plants and raise many additional intriguing aspects of gene regulation. For example, chromatin contact mapping suggests that like in animals, gene expression can be influenced by multiple regulatory regions and that conversely, an individual regulatory region can modulate multiple genes (Wang et al., 2017; Ricci et al., 2019; Gasperini et al., 2020). Understanding this complexity will likely shed light on prior genetic data and assist with future engineering efforts.

## Prospects for Mining Regulatory Diversity in Existing Germplasm

*De novo* whole genome assembly is becoming wide available opening the door for mining regulatory diversity among not only many different plant species, but also closely related inbred lines, accessions, and varieties (Tao et al., 2019; Danilevicz et al., 2020). Such pan-genome collections allow for identification of regulatory variants including both coding and expression alleles including those associated with gene presence/absence, copy number variation, SNPs, indels, and structural variation, and are likely to be highly informative (Darracq et al., 2018; Sun et al., 2018; Gao et al., 2019; Yang et al., 2019a,b; Zhou et al., 2019; Alonge et al., 2020; Song et al., 2020). Similarly, understanding regulatory divergence among sub-genomes in polyploidy species is another exciting yet challenging prospect (Bao et al., 2019). Annotation of both conserved and accession-specific functional elements within these assemblies will likely require both empirical and machine learning based techniques (Michael and VanBuren, 2020). Among these annotation efforts, cataloging and characterizing CREs and individual TF binding events in plant genomes will be essential for understanding transcriptional and phenotypic variation. Much like the genetic maps and gene maps that have guided plant molecular genetics research for the past several decades, we envision that physical maps of annotated non-coding regulatory regions and CREs will be highly useful for both basic research and precision plant breeding. The generation of species-specific “genomic navigation systems” could transform research in much the same way that cellular navigation systems have enabled expanded and more efficient travel in everyday life. Ultimately, the ability to use CRISPR-based technologies to edit specific regulatory elements and alter transcriptional outputs offers great promise for engineering desirable traits (Rodríguez-Leal et al., 2017; Eshed and Lippman, 2019), providing new ways to increase genetic gain and affording a broader spectrum of genetic variation than what is seen in nature, ultimately transforming our approach to crop improvement.

## Author Contributions

All authors listed have made a substantial, direct and intellectual contribution to the work, and approved it for publication.

### Conflict of Interest

The authors declare that the research was conducted in the absence of any commercial or financial relationships that could be construed as a potential conflict of interest.
